# Supplementary material for: An Association between OXPHOS-Related Gene Expression and Malignant Hyperthermia Susceptibility in Human Skeletal Muscle Biopsies
Source: Int J Mol Sci. 2024 Mar 20;25(6):3489. doi: 10.3390/ijms25063489 (PMC10970753; doi:10.3390/ijms25063489)
Supplement: Supplementary file 1 [file ijms-25-03489-s001.zip › Supplemental Table S1- OXPHOS genes.docx]

**Gene ID log2FC lfcSE adjusted p-value hgnc_symbol**

**ENSG00000151366 -0.72 0.18 3.53E-02 *NDUFC2***

**ENSG00000259112 -0.75 0.20 5.30E-02 *NDUFC2-KCTD14***

**ENSG00000174886 -0.82 0.24 6.27E-02 *NDUFA11***

**ENSG00000165264 -0.60 0.18 6.63E-02 *NDUFB6***

**ENSG00000004779 -0.66 0.21 7.53E-02 *NDUFAB1***

**ENSG00000189043 -0.53 0.17 7.71E-02 *NDUFA4***

**ENSG00000110717 -0.81 0.27 8.20E-02 *NDUFS8***

**ENSG00000178057 -0.73 0.24 8.21E-02 *NDUFAF3***

**ENSG00000186010 -0.73 0.24 8.21E-02 *NDUFA13***

**ENSG00000131495 -0.74 0.25 8.48E-02 *NDUFA2***

**ENSG00000213619 -0.72 0.25 8.50E-02 *NDUFS3***

**ENSG00000139180 -0.52 0.18 8.56E-02 *NDUFA9***

**ENSG00000267855 -0.67 0.23 8.71E-02 *NDUFA7***

**ENSG00000115286 -0.67 0.23 8.83E-02 *NDUFS7***

**ENSG00000167792 -0.62 0.22 8.98E-02 *NDUFV1***

**ENSG00000224877 -0.68 0.24 8.98E-02 *NDUFAF8***

**ENSG00000099795 -0.73 0.26 9.00E-02 *NDUFB7***

**ENSG00000185633 -0.69 0.25 9.73E-02 *NDUFA4L2***

**Supplemental Table 1.** Differentially expressed genes encoding complex I in the MHN vs MHS (baseline) comparison. The list of differentially expressed genes encoding for mitochondrial complex I, generated from DESeq2 comparing MHN (n=4) and MHS (n=8) controls at baseline, displayed in order of adjusted p-value.
