# Supplementary material for: An Association between OXPHOS-Related Gene Expression and Malignant Hyperthermia Susceptibility in Human Skeletal Muscle Biopsies
Source: Int J Mol Sci. 2024 Mar 20;25(6):3489. doi: 10.3390/ijms25063489 (PMC10970753; doi:10.3390/ijms25063489)
Supplement: Supplementary file 1 [file ijms-25-03489-s001.zip › Supplemental Table S2 - Upregulated GO MHS baseline.docx]

| **MHN vs MHS (upregulated genes) Gene ontology results** | | | | |
| --- | --- | --- | --- | --- |
| **Biological Process** | **P-value** | **Adjusted p-value** | **Odds Ratio** | **Combined score** |
| mRNA splicing, via spliceosome (GO:0000398) | 1.33E-08 | 1.93E-05 | 6.73E+00 | 1.22E+02 |
| RNA splicing, via transesterification reactions with bulged adenosine as nucleophile (GO:0000377) | 2.82E-08 | 2.05E-05 | 6.87E+00 | 1.19E+02 |
| mRNA processing (GO:0006397) | 4.71E-08 | 2.28E-05 | 6.11E+00 | 1.03E+02 |
| Fat cell differentiation (GO:0045444) | 2.17E-05 | 7.90E-03 | 1.20E+01 | 1.29E+02 |
| RNA splicing (GO:0008380) | 5.08E-05 | 1.48E-02 | 8.02E+00 | 7.93E+01 |
|  |  |  |  |  |
| **Molecular Function** | **P-value** | **Adjusted p-value** | **Odds Ratio** | **Combined score** |
| RNA binding (GO:0003723) | 1.54E-11 | 3.11E-09 | 3.80E+00 | 9.47E+01 |
| mRNA binding (GO:0003729) | 1.17E-05 | 1.18E-03 | 5.08E+00 | 5.77E+01 |
| Cytoskeleton-nuclear membrane anchor activity (GO:0140444) | 9.37E-04 | 4.73E-02 | 6.80E+01 | 4.74E+02 |
| DNA insertion or deletion binding (GO:0032135) | 9.37E-04 | 4.73E-02 | 6.80E+01 | 4.74E+02 |
| Pre-mRNA 3'-splice site binding (GO:0030628) | 1.40E-03 | 5.64E-02 | 5.10E+01 | 3.35E+02 |
|  |  |  |  |  |
| **Cellular Component** | **P-value** | **Adjusted p-value** | **Odds Ratio** | **Combined score** |
| Nuclear lumen (GO:0031981) | 2.00E-08 | 2.84E-06 | 4.06E+00 | 7.20E+01 |
| U2-type spliceosomal complex (GO:0005684) | 2.24E-07 | 1.13E-05 | 1.19E+01 | 1.82E+02 |
| Nucleolus (GO:0005730) | 2.38E-07 | 1.13E-05 | 3.76E+00 | 5.73E+01 |
| Intracellular non-membrane-bounded organelle (GO:0043232) | 1.02E-06 | 3.63E-05 | 2.99E+00 | 4.13E+01 |
| Nucleus (GO:0005634) | 5.52E-05 | 1.57E-03 | 1.85E+00 | 1.82E+01 |

**Supplemental Table S2. MHN vs MHS Gene ontology results for upregulated genes.** A list of the top five enriched biological process, molecular function, and cellular component ontology terms generated using genes upregulated in the MHS (pre-IVCT) phenotype. Terms are displayed in order of adjusted p-value (Statistically significant terms are defined as those with adjusted p-value < 0.05).
