# Supplementary material for: An Association between OXPHOS-Related Gene Expression and Malignant Hyperthermia Susceptibility in Human Skeletal Muscle Biopsies
Source: Int J Mol Sci. 2024 Mar 20;25(6):3489. doi: 10.3390/ijms25063489 (PMC10970753; doi:10.3390/ijms25063489)
Supplement: Supplementary file 1 [file ijms-25-03489-s001.zip › Supplemental Table S3 - Pathway analysis MHSh and MHShc.docx]

| **MHN vs MHS_h_ Pathway Analysis** | | | | |
| --- | --- | --- | --- | --- |
|  |  |  |  |  |
| **Upregulated pathway hallmarks** | **P-value** | **Adjusted p-value** | **Odds Ratio** | **Combined score** |
| Myc Targets V1 | 6.09E-07 | 2.62E-05 | 4.99 | 71.44 |
| mTORC1 Signaling | 9.30E-04 | 2.00E-02 | 3.29 | 22.98 |
| Protein Secretion | 7.29E-03 | 1.04E-01 | 3.74 | 18.38 |
| G2-M Checkpoint | 9.66E-03 | 1.04E-01 | 2.65 | 12.29 |
| E2F Targets | 2.68E-02 | 1.92E-01 | 2.34 | 8.46 |
|  |  |  |  |  |
| **Downregulated pathway hallmarks** | **P-value** | **Adjusted p-value** | **Odds Ratio** | **Combined score** |
| Oxidative Phosphorylation | 3.80E-27 | 1.90E-25 | 7.18 | 436.58 |
| Adipogenesis | 3.70E-07 | 9.24E-06 | 3.03 | 44.92 |
| DNA Repair | 1.03E-05 | 1.72E-04 | 3.02 | 34.67 |
| Myc Targets V1 | 4.17E-04 | 5.21E-03 | 2.26 | 17.59 |
| Glycolysis | 9.65E-04 | 9.65E-03 | 2.16 | 14.97 |
| **MHN vs MHS_hc_ Pathway Analysis** | | | | |
|  |  |  |  |  |
| **Upregulated pathway hallmarks** | **P-value** | **Adjusted p-value** | **Odds Ratio** | **Combined score** |
| TNF-alpha Signaling via NF-kB | 2.07E-09 | 5.17E-08 | 29.42 | 588.35 |
| UV Response Up | 8.93E-06 | 1.12E-04 | 20.88 | 242.80 |
| Hypoxia | 2.79E-05 | 2.32E-04 | 16.35 | 171.50 |
| Cholesterol Homeostasis | 3.18E-04 | 1.99E-03 | 25.47 | 205.13 |
| IL-6/JAK/STAT3 Signaling | 5.12E-04 | 2.56E-03 | 21.52 | 163.04 |
|  |  |  |  |  |
|  |  |  |  |  |
| **Downregulated pathway hallmarks** | **P-value** | **Adjusted p-value** | **Odds Ratio** | **Combined score** |
| Oxidative Phosphorylation | 1.52E-02 | 9.13E-02 | 11.75 | 49.20 |
| TGF-beta Signaling | 5.01E-02 | 1.50E-01 | 20.89 | 62.54 |
| DNA Repair | 1.33E-01 | 1.74E-01 | 7.39 | 14.90 |
| KRAS Signaling Up | 1.74E-01 | 1.74E-01 | 5.52 | 9.66 |
| Myc Targets V1 | 1.74E-01 | 1.74E-01 | 5.52 | 9.66 |

**Supplemental Table S3. Pathway analysis from MHN vs MHS_h_ and MHN vs MHS_hc_ comparisons**. Terms are displayed in order of adjusted p-value (Statistically significant terms are defined as those with adjusted p-value < 0.05).
