# Supplementary material for: An Association between OXPHOS-Related Gene Expression and Malignant Hyperthermia Susceptibility in Human Skeletal Muscle Biopsies
Source: Int J Mol Sci. 2024 Mar 20;25(6):3489. doi: 10.3390/ijms25063489 (PMC10970753; doi:10.3390/ijms25063489)
Supplement: Supplementary file 1 [file ijms-25-03489-s001.zip › Supplemental Table S4 - GO analysis MHSh and MHShc.docx]

| **Gene ontology analyses for the MHN vs MHS_h_ comparison** | | | **Gene ontology analyses for the MHN vs MHS_hc_ comparison** | | |
| --- | --- | --- | --- | --- | --- |
|  | **Upregulated terms** | **Adjusted p-value** |  | **Upregulated terms** | **Adjusted p-value** |
| **(GO_BP) Biological Process** | mRNA splicing, via spliceosome (GO:0000398) | 3.29E-09 | **(GO_BP) Biological Process** | Positive regulation of acute inflammatory response (GO:0002675) | 0.33 |
|  | RNA splicing, via transesterification reactions with bulged adenosine as nucleophile (GO:0000377) | 4.54E-09 |  | Fat cell differentiation (GO:0045444) | 0.34 |
|  | mRNA processing (GO:0006397) | 1.40E-08 |  | Regulation of inflammatory response (GO:0050727) | 0.37 |
|  | RNA metabolic process (GO:0016070) | 7.38E-05 |  | Regulation of fat cell differentiation (GO:0045598) | 0.37 |
|  | RNA processing (GO:0006396) | 3.84E-04 |  | Regulation of vascular associated smooth muscle cell migration (GO:1904752) | 0.45 |
| **(GO_MF) Molecular Function** | RNA binding (GO:0003723) | 5.41E-16 | **(GO_MF) Molecular Function** | Oxidoreductase activity, acting on single donors with incorporation of molecular oxygen, incorporation of two atoms of oxygen (GO:0016702) | 0.75 |
|  | mRNA binding (GO:0003729) | 2.85E-07 |  | Manganese ion binding (GO:0030145) | 1.00 |
|  | Translation initiation factor activity (GO:0003743) | 1.08E-03 |  | Clathrin heavy chain binding (GO:0032050) | 1.00 |
|  | Translation factor activity, RNA binding (GO:0008135) | 6.86E-03 |  | Primary amine oxidase activity (GO:0008131) | 1.00 |
|  | Proteasome-activating atpase activity (GO:0036402) | 2.45E-02 |  | RNA polymerase II core promoter proximal region sequence-specific DNA binding (GO:0000978) | 1.00 |
| **(GO_CC) Cellular Component** | Nucleolus (GO:0005730) | 1.70E-07 | **(GO_CC) Cellular Component** | Lipid droplet (GO:0005811) | 1.00 |
|  | Spliceosomal complex (GO:0005681) | 3.86E-07 |  | Endoplasmic reticulum lumen (GO:0005788) | 1.00 |
|  | Nuclear body (GO:0016604) | 1.04E-04 |  | Extrinsic component of external side of plasma membrane (GO:0031232) | 1.00 |
|  | Nuclear speck (GO:0016607) | 7.09E-04 |  | Nuclear speck (GO:0016607) | 1.00 |
|  | Spindle pole (GO:0000922) | 1.86E-03 |  | Nuclear body (GO:0016604) | 1.00 |
|  |  |  |  |  |  |
|  |  |  |  |  |  |
|  | **Downregulated terms** | **Adjusted p-value** |  | **Downregulated terms** | **Adjusted p-value** |
| **(GO_BP) Biological Process** | Respiratory electron transport chain (GO:0022904) | 1.40E-15 | **(GO_BP) Biological Process** | Heart trabecula formation (GO:0060347) | 1.00 |
|  | Mitochondrial ATP synthesis coupled electron transport (GO:0042775) | 1.63E-15 |  | Membrane invagination (GO:0010324) | 1.00 |
|  | Mitochondrial respiratory chain complex assembly (GO:0033108) | 1.17E-13 |  | Negative regulation of establishment of protein localization (GO:1904950) | 1.00 |
|  | Mitochondrial translational elongation (GO:0070125) | 1.68E-13 |  | SMAD protein complex assembly (GO:0007183) | 1.00 |
|  | Cellular protein metabolic process (GO:0044267) | 3.45E-13 |  | Amyloid fibril formation (GO:1990000) | 1.00 |
| **(GO_MF) Molecular Function** | NADH dehydrogenase (ubiquinone) activity (GO:0008137) | 1.54E-08 | **(GO_MF) Molecular Function** | FK506 binding (GO:0005528) | 0.10 |
|  | NADH dehydrogenase (quinone) activity (GO:0050136) | 7.68E-09 |  | Peptidyl-prolyl cis-trans isomerase activity (GO:0003755) | 0.34 |
|  | Hydrogen ion transmembrane transporter activity (GO:0015078) | 9.77E-06 |  | Type I transforming growth factor beta receptor binding (GO:0034713) | 1.00 |
|  | NADH dehydrogenase activity (GO:0003954) | 2.13E-04 |  | Phosphatidylinositol-5-phosphate binding (GO:0010314) | 1.00 |
|  | Atpase activity, coupled to transmembrane movement of ions, rotational mechanism (GO:0044769) | 1.94E-04 |  | Activin binding (GO:0048185) | 1.00 |
| **(GO_CC) Cellular Component** | Mitochondrion (GO:0005739) | 8.61E-31 | **(GO_CC) Cellular Component** | Mitochondrial proton-transporting ATP synthase complex (GO:0005753) | 0.09 |
|  | Mitochondrial inner membrane (GO:0005743) | 1.10E-28 |  | Mitochondrion (GO:0005739) | 0.50 |
|  | Mitochondrial respiratory chain complex I (GO:0005747) | 6.23E-10 |  | Mitochondrial inner membrane (GO:0005743) | 0.58 |
|  | Mitochondrial matrix (GO:0005759) | 1.37E-08 |  | Mitochondrial proton-transporting ATP synthase complex, coupling factor F(o) (GO:0000276) | 0.59 |
|  | Mitochondrial large ribosomal subunit (GO:0005762) | 4.97E-08 |  | Early phagosome (GO:0032009) | 0.74 |

**Supplemental Table S4. Gene ontology analysis for the MHN vs MHS_h_ and MHN vs MHS_hc_ comparisons.** A list of the enriched biological process, molecular function, and cellular component ontology terms listed in order of adjusted p-value (significance defined by p-value <0.05).
