# Supplementary material for: An Association between OXPHOS-Related Gene Expression and Malignant Hyperthermia Susceptibility in Human Skeletal Muscle Biopsies
Source: Int J Mol Sci. 2024 Mar 20;25(6):3489. doi: 10.3390/ijms25063489 (PMC10970753; doi:10.3390/ijms25063489)
Supplement: Supplementary file 1 [file ijms-25-03489-s001.zip › Supplemental Table S5 - TaqMan assay list.docx]

| **TaqMan RT-qPCR assays (OXPHOS genes)** | | | | | |
| --- | --- | --- | --- | --- | --- |
| **Applied Biosystems^TM^ Assay ID** | **Gene** | **Log2FC** | **Adjusted p-value** | **Rationale** | **Mitochondrial component** |
| Hs00910071_g1 | *ATP5MD* | -0.722 | 0.0530 | MHN vs MHS (baseline): Reduced expression in MHS Top 5 lowest adjusted p-values | Complex V |
| Hs00370770_m1 | *MALSU1* | -0.628 | 0.0530 |  | Mitochondrial Assembly of Ribosomal Large Subunit 1 |
| Hs00190012_m1 | *NDUFC2* | -0.719 | 0.0353 |  | Complex I |
| Hs05050186_s1 | *COQ6* | -0.659 | 0.0353 |  | Coenzyme Q6, Monooxygenase |
| Hs00967250_m1 | *MPC2* | -0.445 | 0.0663 |  | Mitochondrial Pyruvate Carrier 2 |
| Hs00418300_m1 | *NDUFA11* | -0.818 | 0.0627 | MHN vs MHS (baseline): Reduced expression in MHS Top 5 log2FC | Complex I |
| Hs01042479_m1 | *SDHB* | -0.515 | 0.1028 |  | Complex II |
| Hs03054640_g1 | *UQCC3* | -0.889 | 0.0930 |  | Complex III |
| Hs0017909_m1 | *COX8A* | -0.821 | 0.0657 |  | Complex IV |
| Hs00961521_m1 | *ATP5F1D* | -0.836 | 0.0943 |  | Complex V |

**Supplemental Table S5. TaqMan RT-qPCR assays for RNAseq validation with rationale for gene selection.**
