# Supplementary material for: An Association between OXPHOS-Related Gene Expression and Malignant Hyperthermia Susceptibility in Human Skeletal Muscle Biopsies
Source: Int J Mol Sci. 2024 Mar 20;25(6):3489. doi: 10.3390/ijms25063489 (PMC10970753; doi:10.3390/ijms25063489)
Supplement: Supplementary file 1 [file ijms-25-03489-s001.zip › Supplemental Table S6 - Samples selected for RT-qPCR.docx]

| **MHS_h_ Patient no (n=18)** | **age at biopsy** | **Sex** | **Contracture @ 2% halothane** | **RYR1 variant** | **CACNA1S variant** |
| --- | --- | --- | --- | --- | --- |
| 13 | 30 | Female | 0.2 | - | - |
| 14 | 32 | Female | 0.2 | - | - |
| 15 | 58 | Female | 0.35 | c.11315G>A | - |
| 16 | 45 | Female | 0.2 | - | c.4060A>T |
| 17 | 27 | Female | 0.55 | c.14210G>A | - |
| 18 | 27 | Female | 0.2 | c.7268T>A | - |
| 19 | 39 | Female | 0.25 | c.6785G>A | - |
| 20 | 44 | Female | 0.2 | c.251C>T | - |
| 21 | 15 | Female | 0.2 | c.5183C>T | - |
| 22 | 18 | Male | 0.2 | - | - |
| 23 | 56 | Male | 0.2 | - | c.530C>T |
| 24 | 43 | Male | 0.2 | c.5183C>T | - |
| 25 | 28 | Male | 0.2 | - | - |
| 26 | 38 | Male | 0.2 | - | - |
| 27 | 31 | Male | 0.2 | - | - |
| 28 | 53 | Male | 0.2 | - | - |
| 29 | 61 | Male | 0.2 | - | - |
| 30 | 15 | Male | 0.2 | c.7816T>A | - |
|  |  |  |  |  |  |
| Female | age range (15-58) |  |  |  |  |
| Male | age range (15-61) |  |  |  |  |

|  |  |  |
| --- | --- | --- |

| **MHS_hc_ Patient no (n=18)** | **age at biopsy** | **Sex** | **Contracture @ 2% halothane** | **RYR1 variant** | **CACNA1S variant** |
| --- | --- | --- | --- | --- | --- |
| 31 | 11 | Female | 1.85 | c.14545G>A | - |
| 32 | 19 | Female | 2.3 | c.14449A>T | - |
| 33 | 14 | Female | 2.1 | c.7292A>T | - |
| 34 | 13 | Female | 1.2 | c.7063C>T | - |
| 35 | 18 | Female | 0.35 | c.3166G>C | - |
| 36 | 12 | Female | 1.6 | c.1021G>A | - |
| 37 | 16 | Female | 0.9 | c.1021G>A | - |
| 38 | 52 | Female | 2.3 | c.455C>A | - |
| 39 | 34 | Female | 1.5 | c.6612C>G | - |
| 40 | 16 | Male | 1.5 | c.14201G>A | - |
| 41 | 28 | Male | 0.55 | c.11132C>T | - |
| 42 | 30 | Male | 0.2 | - | - |
| 43 | 51 | Male | 0.85 | c.8729C>T | - |
| 44 | 12 | Male | 0.25 | c.11958C>G | - |
| 45 | 30 | Male | 2.55 | c.6612C>G | - |
| 46 | 29 | Male | 1.45 | c.1202G>A | - |
| 47 | 55 | Male | 0.45 | c.9758T>C | - |
| 48 | 57 | Male | 0.85 | c.7879G>A | - |
|  |  |  |  |  |  |
| Female | age range (12-52) |  |  |  |  |
| Male | age range (12-57) |  |  |  |  |

**Supplemental Table S6. Additional MHS_h_ and MHS_hc_ samples selected for RNAseq validation work using RT-qPCR.**
