# Supplementary figures and images for: An Association between OXPHOS-Related Gene Expression and Malignant Hyperthermia Susceptibility in Human Skeletal Muscle Biopsies
Source: Int J Mol Sci. 2024 Mar 20;25(6):3489. doi: 10.3390/ijms25063489 (PMC10970753; doi:10.3390/ijms25063489)

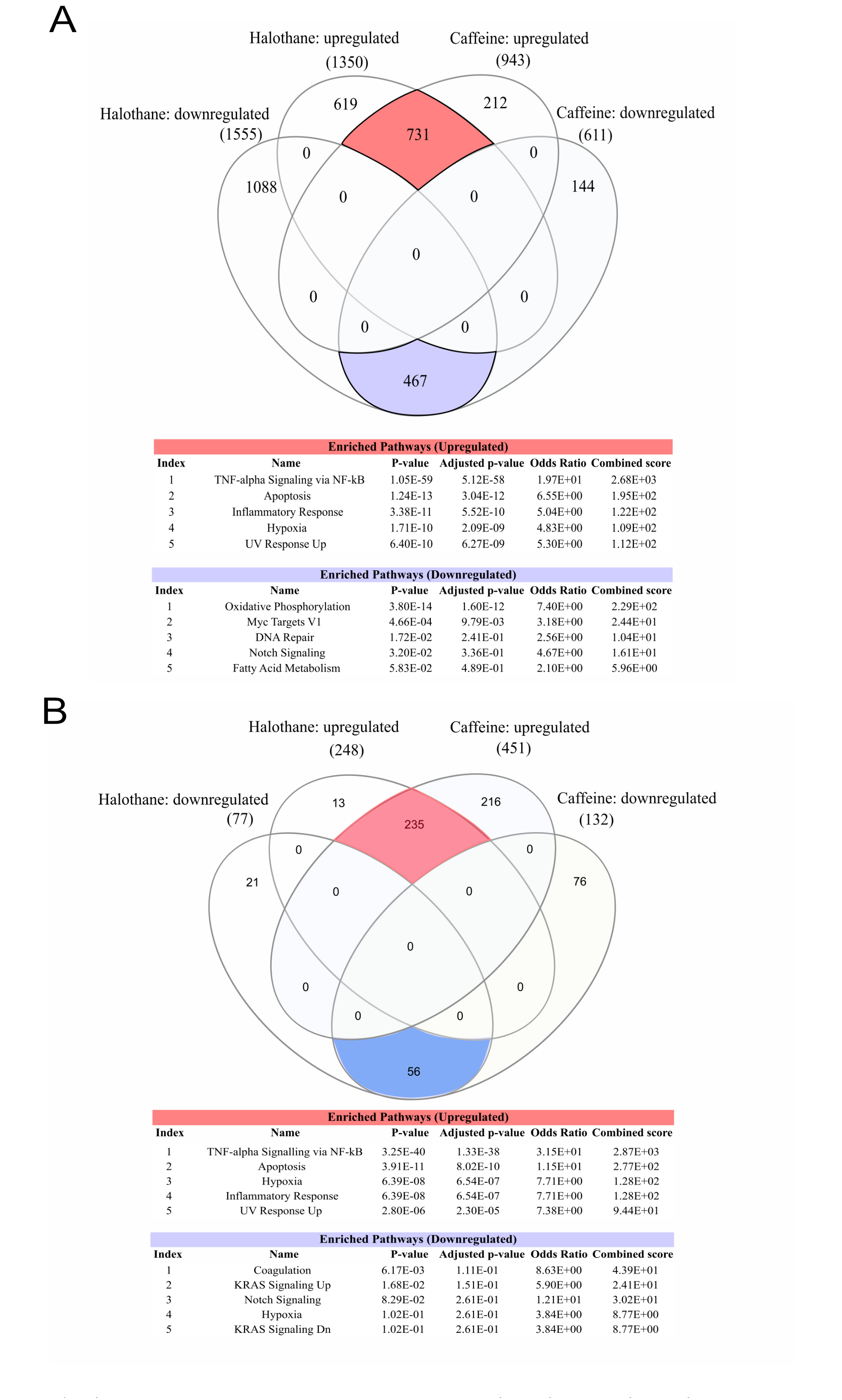

Supplement: Supplementary file 1 [file ijms-25-03489-s001.zip › Supplementary Figure S1.tiff]
